# The Impact of Stopping Risk Assessment Checklists at a Specialist Personality Disorder Unit

**DOI:** 10.7759/cureus.33935

**Published:** 2023-01-18

**Authors:** Adelaide C Yue, Alasdair W Philbey, Owen A Crawford, Jorge Zimbron

**Affiliations:** 1 School of Clinical Medicine, University of Cambridge, Cambridge, GBR; 2 School of Clinical Medicine, Sandwell and West Birmingham Hospitals NHS Trust, Birmingham, GBR; 3 Springbank Ward - Inpatient Personality Disorders, Cambridgeshire and Peterborough NHS Foundation Trust, Cambridge, GBR

**Keywords:** psychiatric risk, face-to-face interview, leave protocol, risk assessment, borderline personality disorder, emotionally unstable personality disorder

## Abstract

Aim

This study was conducted in Springbank Ward, a specialist ward for patients with emotionally unstable personality disorder, based in Cambridge, United Kingdom. We aimed to assess any change in incident frequency following the introduction of a new protocol for leaving the ward, in which patients are offered an optional conversation with staff in place of a formal risk assessment checklist. We also aimed to assess patient and staff perceptions of the change.

Methods

We used data routinely collected by Springbank Ward to compare incident frequency in the year before and after the change in protocol. We conducted structured interviews with patients and staff to obtain qualitative data on the new protocol and used thematic analysis to interpret the interview data.

Results

There were 466 incidents during the period before the change in protocol and 408 incidents in the period after. Adjusted for occupancy rate, there was no statistically significant difference in the frequency of incidents. Patients and staff were generally satisfied with the new protocol, with an average satisfaction rating of 4.1 out of 5. Thematic analysis generated five main themes: 'taking ownership', 'autonomy versus restriction', 'staff-patient interaction', 'staff expertise' and 'protocol efficiency'.

Conclusions

Our study reveals high satisfaction with the new way risk is assessed and managed for patients leaving Springbank Ward, with an appreciation for its holistic and minimally restrictive approach. This was achieved without significantly increasing incident frequency.

## Introduction

Emotionally unstable personality disorder (EUPD), also known as borderline personality disorder (BPD), is characterised by features such as emotional dysregulation, impulsivity and unstable relationships [1]. Due to the increased risk of incidents of self-harm and suicide for patients with EUPD relative to the general population [2], mental health units often adopt restrictive, defensive strategies to minimise these risks, as opposed to recovery-oriented approaches that use positive risk-taking to facilitate the independence and skills required for life in the community [3-5]. Defensive practice often includes the use of risk assessment checklists before allowing patients to leave wards, despite limited evidence that these checklists reduce the incidence of self-harm, suicide or violence [6,7]. Previous research on risk assessment has found that mental health professionals have mixed views on protocols involving checklists. While they may provide a sense of security for some clinicians [8] and be used to facilitate decision-making [9], they can be overly reductive [10] and may impair therapeutic relationships [9]. A documentary analysis of risk assessment protocols found little incorporation of positive risk-taking, raising questions about their impact on the recovery process [11].

Springbank Ward is a specialist EUPD inpatient unit with an ethos based on supporting patients to live autonomously with minimal restrictions. Previously, prior to a patient leaving the ward temporarily (for example, to go to the supermarket), it was compulsory for staff to complete a formal risk assessment checklist. Crawford et al. conducted interviews with patients and staff in Springbank Ward and found that most were in favour of a more flexible and holistic approach when assessing risk [12]. As a result of the findings from this service evaluation, the protocol for patients leaving the ward has been altered to remove the checklist component. Instead, patients are now offered a 1:1 conversation with a staff member before they leave the ward. This provides the patient with an opportunity to seek support from staff. The new protocol was developed and discussed at several team meetings before its introduction and became part of the induction programme for new ward staff. The details of both the former and current protocols have been published previously [12], but are summarised in Appendix 1. Consistent with best practice quality improvement, this study aimed to re-evaluate patient and staff perspectives on the new leave protocol to ensure the changes made have had the desired effect of making the ward a safer and more therapeutic environment. It also aimed to quantitatively assess any change in incident frequency after the introduction of the new protocol.

A summary of this work was previously presented as an oral presentation at the British and Irish Group for the Study of Personality Disorder 2022 Conference and as a poster at the Royal College of Psychiatrists 2022 International Congress.

## Materials and methods

Study setting

Springbank Ward is a 12-bed inpatient recovery unit for women, transgender people, and non-binary individuals between the ages of 18 and 65 with a diagnosis of EUPD. Patients typically are admitted for a one-year treatment course.

Data collection and analysis

Investigating the Effect on Incident Frequency

We used routinely collected data relating to patient incidents, from one year before to one year after 2 March 2020, when the new leave protocol was implemented. Incidents are defined as points where there is a potential for harm to occur to a patient or others due to the patient’s involvement. They include events such as self-harm and suicide attempts.

We sorted incidents into categories based on the outcome: ‘No Harm’; ‘Low (Minimal Harm)’; ‘Moderate (Short Term Harm)’; or ‘Severe (Long Term Harm)'. Incidents were also sorted according to whether they occurred on or off the ward. Incidents were then categorised based on whether they occurred prior to 2 March 2020 or after.

To further investigate how the change in protocol affected incident frequency over time, we used the NHS Statistical Process Control (SPC) tool, a standardised tool used in the NHS to track changes following interventions [13]. Only incidents causing harm were included in this, as these were considered the most important in relation to patient safety. As patient numbers vary across the year, the data were standardised to account for variable occupancy rates. Occupancy rates were collected as bed-days per month, with one bed-day representing one patient spending one day on the ward. The number of incidents causing harm per month was standardised by dividing by the number of occupied bed-days per month. The NHS SPC tool was then used to produce a graph of incidents per 100 occupied bed-days over time. It was also used to search for significant deviations in the data (with significance defined as 3 standard deviations above or below the mean).

Investigating Patient and Staff Perceptions of the New Protocol

Qualitative data were obtained through structured interviews with staff who assessed risk (nurses and psychiatrists) and patients. Patients and staff were selected for interview by opportunistic sampling and provided written informed consent to participate. Interviews were conducted between 9 March 2021 and 19 March 2021. Each interviewee rated satisfaction with the new protocol and responded to a series of open-ended questions (see Appendix 2). Responses were transcribed into an electronic form at the time of the interview.

Thematic analysis was used to analyse interview data. AY and OC independently analysed the interview transcripts to categorise the data into preliminary themes. AY and OC then discussed any discrepancies to reach a unified set of themes, subthemes and codes.

## Results

Effect on incident frequency

There were 466 incidents during the period before the change in protocol and 408 incidents in the period after. Table 1 shows the number of incidents before and after the change in protocol by location and degree of harm. No incidents were categorised as severe during the period of analysis.

**Table 1:** Number of incidents before and after the change in risk assessment protocol

Incident location	Degree of harm	Before new protocol	After new protocol	Change (%)
On the ward	No harm	104	87	-16.3%
	Low (Minimal harm)	253	266	+5.1%
	Moderate (Short term harm)	25	14	-44.0%
	Total	382	367	-3.9%
Off the ward	No harm	24	11	-54.2%
	Low (Minimal harm)	44	28	-36.4%
	Moderate (Short term harm)	16	2	-87.5%
	Total	84	41	-51.2%
On and off the ward	No harm	128	98	-23.4%
	Low (Minimal harm)	297	294	-1.0%
	Moderate (Short term harm)	41	16	-61.0%
	Total	466	408	-12.4%

**Figure 1:**
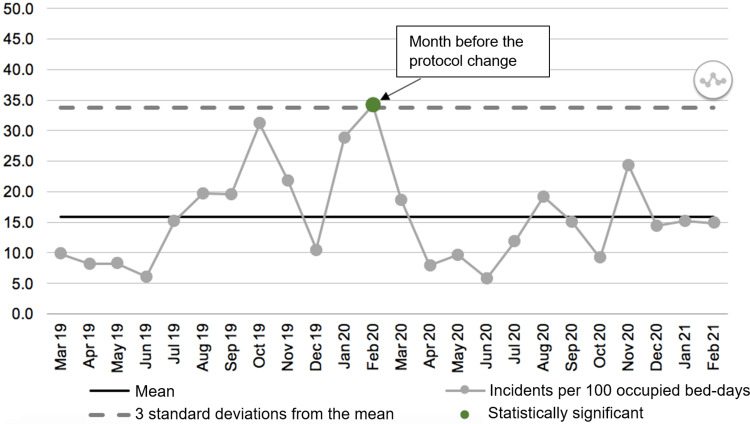
Incidents per 100 occupied bed-days a year before and after the change in protocol

Tracking the frequency of incidents causing harm over time, a year before and after the protocol change, there was no statistically significant difference in the frequency of incidents, with the exception of February 2020, the month before the protocol change. For this month, there was a number of incidents per 100 occupied bed-days significantly above the mean (Figure 1).

Interview results

Interviewee demographics are shown in Table 2.

**Table 2:** Interviewee demographics

		Patients (*N*=9)	Staff (*N*=8)
Gender	Female (*n*, %)	7 (78)	5 (63)
	Non-binary (*n*, %)	1 (11)	0 (0)
	Male (*n*, %)	1 (11)	3 (37)
Age in years: *Mean *(range)		23 (18–29)	38 (29–54)

**Figure 2:**
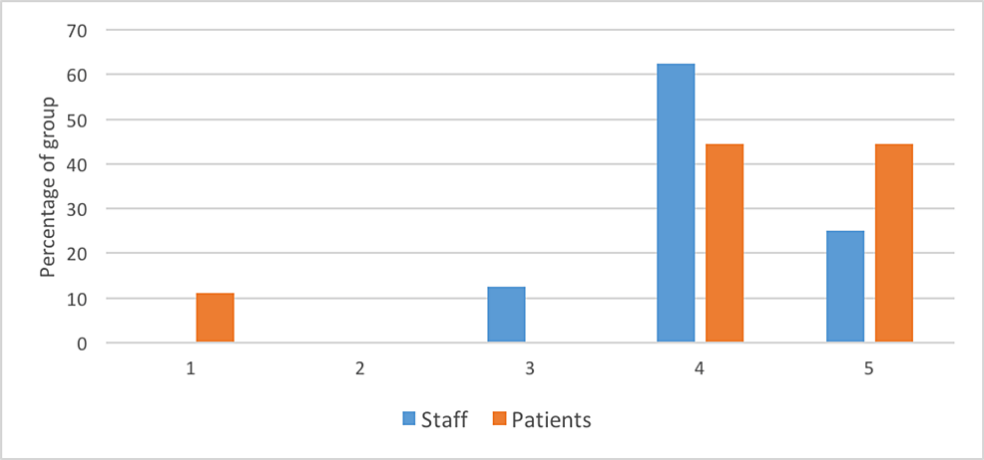
Patient and staff satisfaction ratings

Figure 2 shows the staff and patient responses to the question “On a scale of 1-5, where 1 is not satisfied at all, and 5 is completely satisfied, how satisfied are you with the process of leaving the ward?” Mean patient satisfaction was 4.1 and mean staff satisfaction was 4.1. In comparison, the previous protocol obtained a mean satisfaction rating of 2.5 out of 5 from staff and 2.75 out of 5 from patients [12].

Analysis of interview transcripts identified five main themes: taking ownership; autonomy versus restriction; staff-patient interaction; staff expertise; and protocol efficiency.

Taking Ownership

This theme encompassed the concept of patients being supported to take ownership and responsibility for their recovery and was mentioned by eight patients and eight staff members. Most reported that the new leave protocol encouraged patients to be responsible for their safety by initiating conversations with staff if they required support. However, some patients felt that a more structured checklist similar to the previous protocol could assist in identifying areas where support may be needed.

“I think it’s an important part of being at Springbank because Springbank is all about taking responsibility and ownership of your recovery and almost being…having that responsibility of asking for help, asking to talk to someone, is good, because no-one’s a mind reader.” - Patient 2

“I think the fact that having a chat is left up to them provides them with that responsibility of seeking help if they need it.” - Staff 5

“I feel that a less formal checklist would be useful for us, that we can fill out rather than staff filing out, so have I taken the meds I need to…am I feeling ok…” - Patient 7

Staff were viewed as having a facilitating role in helping patients take ownership of their recovery, compared to the previous system in which responsibility was placed more on staff.

“Ultimately it was the nurses making the decision on whether you could go on leave, rather than the patient for the old one, so I think the new one is better in comparison.” - Staff 1

Patients and staff both mentioned that by being encouraged to take responsibility for their safety, patients were more prepared for life after discharge, as they would be responsible for seeking support once living in the community.

“The new system represents the real world, where a patient decides they want to do something and they go and do it, and we're there to help if you need it, but the responsibility for getting help lies with the patient, just like they might phone their mum and say "I'm going out", so it's more of a realistic role.” - Staff 7

“That’s really good practice for when I’m back out in the community, I like the fact I feel like I’ve got my freedom here especially as it’s a long stay ward, I think if the leave policy was more restrictive I’d sort of get used to someone taking responsibility for my safety and when I got out in the community it would be overwhelming to have that freedom again.” - Patient 1

Autonomy Versus Restriction

This theme was covered by seven patients and six staff members and focused on the balance between allowing freedom and restricting patients from leaving the ward. Most considered the new protocol to be minimally restrictive, with the exception of one staff member who thought that patients could be given even more autonomy.

“I like the current system because it gives more leeway into going out and it just gives you freedom and it feels like it’s not a locked ward.” - Patient 4

“The only improvement I would foresee is for patients to have their own keys and let themselves in and out of the ward but that would not happen, because national policy about the functioning of mental health wards in 2021 wouldn't allow it.” - Staff 5

Some patients and staff members perceived the lack of restriction as inherently risky and potentially anxiety-provoking, compared to the sense of security previously provided by a formal risk assessment checklist.

“I think it works most of the time, but there are occasions where it falls down, like…so a patient might be suicidal or intending on self harm, and they refuse 1:1 with a qualified nurse and they're still allowed out anyway.” - Patient 8

“There are a few incidents where patients have self harmed so from my point of view, it’s just, from a staff perspective, they feel quite anxious when letting patients off the ward.” - Staff 6

“It can sometimes be a bit scary and sometimes you want them to stop you, but they don’t.” - Patient 3

However, interviewees also felt that this risk was balanced by the long-term benefit of reducing restrictions and that the system was safe overall.

“It raises staff anxieties, even now, and I’ve been here years, and the other day there was an incident with someone wanting to go out, and we know what could be potentially coming...but you know in the long term you are helping that person, compared to the short run just because you’re anxious as a staff member.” - Staff 4

“They seem to have like a least restrictive practice but keeps us safe at the same time.” - Patient 6

Staff-Patient Interaction

This theme, mentioned by nine patients and seven staff members, encompassed the role of communication and the therapeutic relationship between patients and staff. In general, the current ward environment was considered to promote communication through offering a 1:1 conversation with a staff member, whereas the previous risk assessment checklist limited open conversation. It was felt that patients generally used the 1:1 conversation if they felt they needed support. This contributed to the view that patients were encouraged to take ownership of their safety by this new protocol.

“If you are encouraged to talk to staff before you go and you seem not quite right, they'll say, ‘Are you sure you’re not gonna have a chat?’ And I think that’s much more effective in getting you to tell the truth rather than just going through a tick box thing.” - Patient 1

The importance of building trust between staff and patients in order to encourage open conversation was emphasised. A few patients mentioned they found it difficult to be open with staff and that more structured support would encourage increased communication, particularly early in admission. This was echoed by some staff members, who felt they could take more of a leading role in initiating 1:1 conversations for new patients, with that responsibility gradually being shifted to the patient as the admission progresses.

“If you have a good relationship with the patient…then they’ll say yes to a chat because they want to chat to you.” - Staff 2

“If you are really struggling, like especially at the start of an admission and you're not used to being informal and going out on your own, I think to begin with there should be more support around leaving, like the old form, so you have a chance to express yourself…then as you're further along in admission it can change to something more like [the new protocol] because then you've got the skills to learn how to take trust.” - Patient 9

“I suppose new patients… don’t know and trust us yet, and don’t have a good enough relationship that they feel like they need support when they need it...I suppose for new patients when we give that option of ‘have a chat’, it should be ‘let’s have a chat before we go out’.” - Staff 1

Staff Expertise

This theme, mentioned by four patients and five staff members, focused on the importance of staff experience and skill in helping the protocol function effectively. A prominent subtheme was the use of this expertise to assess risk holistically, as opposed to a reductive checklist. Staff were considered to be continually assessing risk based on patients’ recent behaviour, using their judgment and intuition and prompting 1:1 conversations with patients they believed to be at risk.

“There used to be a formal risk assessment, but I think now the risk assessment is kind of ongoing, so we're constantly being risk assessed in nurses' heads, so if we ask to leave they have a good idea of what our intentions might be, whether they're completely innocent or slightly more dark.” - Patient 8

“I’ve heard that people say ‘Springbank just open the doors and let them out’ when actually there’s a lot of assessment behind letting someone out, it’s the whole day, you analyse how someone is on the ward.” - Staff 1

“[The new protocol] enables you to use your clinical judgment and nursing skills.” - Staff 4

Staff would strongly encourage a 1:1 conversation if they believed a patient to be at risk. The importance of teamwork and communication between staff was emphasised as vital for making appropriate decisions based on perceived risk.

“Sometimes you sort of have to have a 1:1 if you're not viewed as safe, yeah, which has happened to me a few times.” - Patient 7

“I think because it relies so much on health care assistants and everyone else keeping you informed of risk, sometimes if you've been off the ward for a long time...you're reliant on others communicating what’s happened, you're not sure where you're at and someone's handing you a bit of paper and asking if they can go out.” - Staff 7

One perceived limitation of the reliance on staff expertise was the potential difficulty faced by inexperienced staff in adapting to this method of managing risk. This, alongside the minimal restriction, increased staff anxieties. It was also felt that no matter which protocol was in place, staff still had limited insight into patients’ mental states and there was no infallible method of predicting risk.

“There is still a gap of what we’re doing in particular for new staff…I think there’s a risk they’ll assume that’s how we do it at Springbank, and if someone says no to a chat before going off the ward they just take that as verbatim, whereas people who have used the old process have the benefit of knowing why we do it and I suppose the confidence to engage in difficult conversations.” - Staff 1

“I guess it’s just the discomfort that comes with knowing you can ask someone if they want to chat, you know they can decline that, can present very calm and perfectly fine, but otherwise may have other plans…but there is no protocol or checklist that could be made that makes us mind readers.” - Staff 3

Protocol Efficiency

This theme focused on the speed and simplicity of the new protocol and was mentioned by nine patients and four staff members. The new protocol was generally considered to be quicker and more efficient than the previous system. Most patients thought it takes 5-10 minutes between asking to leave and leaving the ward.

“This frees up a lot of nursing time as well to do other things. We used to spend ages having conversations and then writing up...every time someone wants to go out we had to write it up as a note, and it took time away from having other conversations about other things with patients.” - Staff 7

“…Usually takes about 5-10 minutes just from asking depending on how busy the staff are and depending on whether you haven't taken the meds you need to take or if you're waiting for meds to go out, or if you're waiting for a chat or something it can take longer.”- Patient 7

The main reason for the time delay was the staff being busy and, therefore, being unable to sign patients out immediately. One staff member felt that the current protocol still contained unnecessary paperwork such as the box to sign back in.

“Sometimes it’s straight away if staff are free, sometimes it’s up to 20 minutes if no nurse is available, that seems to be the main reason for a delay, because the nurses are busy, and you just have to wait for a nurse to sign you out.” - Patient 1

“The only thing I don't like is the signing back in box, I find it gets used so infrequently that it’s actually probably detrimental, so if we created this system, yet we've created this part of the system that never gets used, why is it still in the system? We have no legal requirement to note when they come back, so...” - Staff 8

## Discussion

Comparison with previous literature

Our results suggest that the new protocol for patients leaving the ward achieved the aims set out by Crawford et al. [12]: the introduction of a protocol with more freedom for patients that encouraged them to take responsibility for their safety, with risk assessment being performed in a more holistic and individualised manner. Patients and staff were, on average, more satisfied with the new protocol than the previous one. In addition, the new protocol was generally perceived to be relatively quick, increasing the amount of time available for staff to perform other duties, whereas the time-consuming nature of the previous protocol's checklist had been highlighted as a major issue [12]. The perceived riskiness of the new protocol in our study echoed previous concerns about relaxing restrictions [12]. However, we have not found evidence of a significant change in incident frequency following the change in protocol.

To our knowledge, there is no other literature in this area specific to adults with EUPD. However, our results reflect research on risk assessment in other psychiatric populations. For example, Muir-Cochrane et al. conducted interviews with staff from an Australian mental health service to investigate their views on risk assessment. These authors highlighted similar themes of holistically assessing risk, promoting positive risk-taking in a supportive environment with clinician oversight, and the importance of communication within the multidisciplinary team [14]. Similarly, Godin’s 2004 paper on risk assessment approached by community mental health nurses found that many advocated a personalised approach to assessment, incorporating background knowledge of the patient and professional intuition. They also expressed that formal checklists were often too simplistic and reductive [10].

Study limitations

In addition to the limitations of interviews conducted in this environment, which have been discussed previously [12], there were a number of additional limitations of our study. It was not possible to determine the influence of confounding factors such as the coronavirus disease 2019 (COVID-19) pandemic. Patients continued to leave the ward to exercise and go to the supermarket, but the range of options available was reduced due to the national lockdowns, which could have decreased incident frequency. The study is also limited by the use of opportunistic sampling, which could have introduced bias into the interview results. It is important to note that our results relate to patients in a specialist unit and may not necessarily be applicable to people with a personality disorder being cared for in a different setting. The impact of similar changes in other settings like acute psychiatric wards remains to be explored.

## Conclusions

After a formal risk assessment checklist was removed from the protocol for patients leaving the ward and replaced with the offering of a conversation with a staff member, there was no statistically significant change in incident frequency. Patients and staff were more satisfied with the new leave protocol. It was generally perceived to facilitate patients in taking ownership of their recovery while allowing staff to holistically assess and manage risk. Overall, this change offers an improvement in patient satisfaction at Springbank Ward without compromising patient safety. There are limitations to the study that mean further research is required, but this is a promising avenue of clinical practice that provides opportunities to create safer and more empowering environments for patients with EUPD.

## Figures and Tables

**Figure 3:**
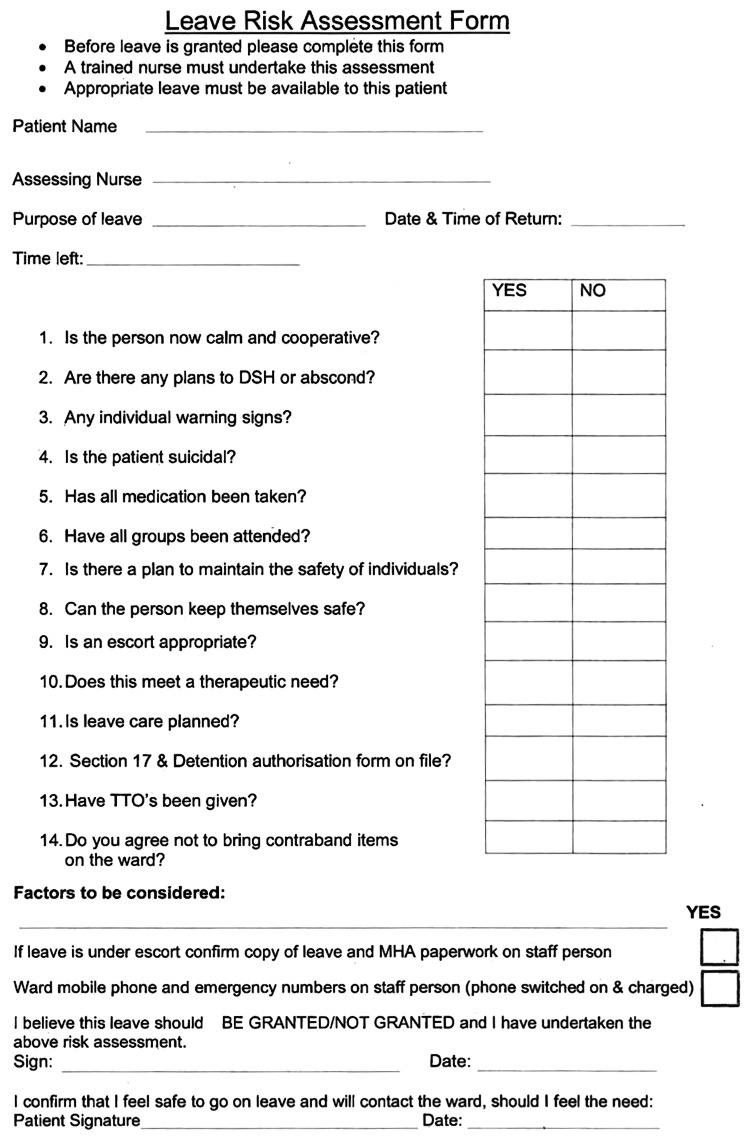
The previous protocol for leaving the ward

**Figure 4:**
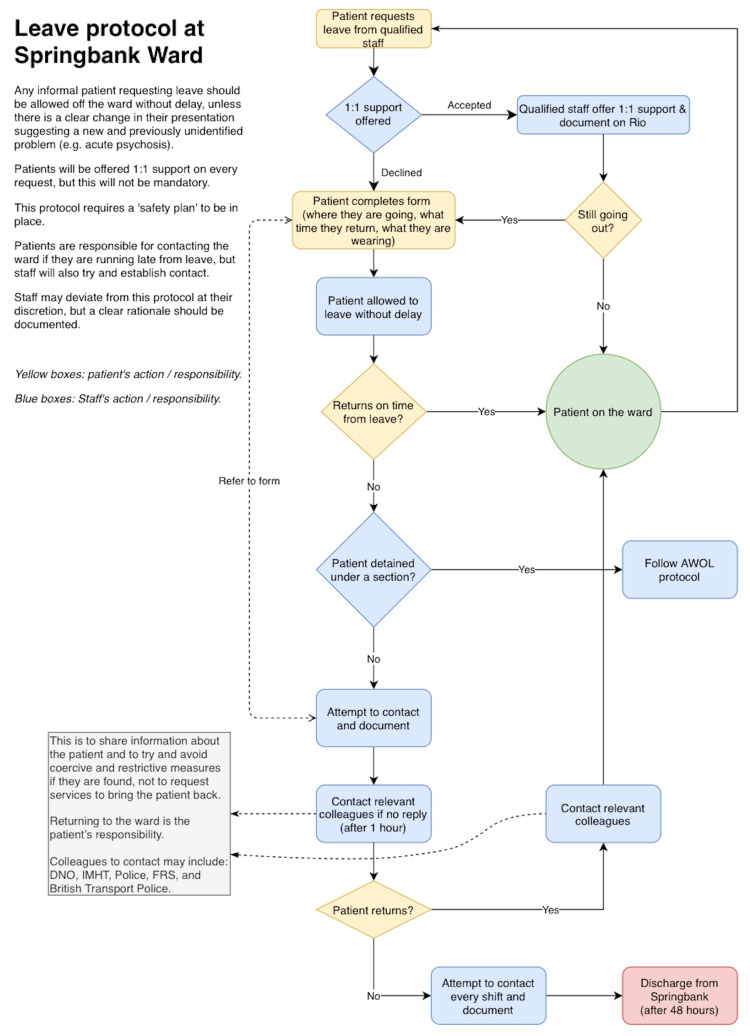
The new protocol for leaving the ward

## Appendices

Appendix 1: previous and new protocols for leaving the ward

The following image shows the previous protocol for patients leaving the ward, a formal risk assessment checklist.

The following image shows the new protocol for patients leaving the ward, in which the formal risk assessment checklist was removed and replaced with an optional 1:1 conversation with a staff member.

Appendix 2: interview questions

The following is the script used to interview patients and staff members:

1. (Staff only; patient demographic information was provided by Springbank Ward) First I would like to collect some demographic data: please may you tell me your ethnicity, gender, age and the month and year you started at Springbank? 2. (Staff only) Please may you tell me if you previously used the old leave protocol with the risk assessment checklist? (Y/N) 3. On a scale of 1-5, where 1 is not satisfied at all, and 5 is completely satisfied, how satisfied are you with the process of leaving the ward? Why? 4. Do you think the current system for leaving the ward could be improved, and if so, how? 5. How often do you (patient interview) / patients (staff interview) use the 1:1 offered before leaving the ward, and why? 6. (Patients only) On average, how long does it take from the time you ask to leave, to leaving the ward, and why? 7. Does the system for leaving the ward encourage patients to take ownership for their actions and safety? What are your thoughts on this? 8. (Patients only) Would you prefer going through a checklist like this one (points to checklist previously used), or do you prefer the current system (points to flowchart of current protocol), and why? 9. (Staff only) If not previously used the old protocol: Would you prefer going through a checklist like this one (points to checklist previously used), or do you prefer the current system (points to flowchart of current protocol), and why? If previously used the old protocol: Comparing this system to the previous protocol with a risk assessment checklist (point to checklist and flowchart of current protocol), do you prefer one or the other, and why?
